# Injection-Locked, Single Frequency, Multi-core Yb-doped Phosphate Fiber Laser

**DOI:** 10.1038/s41598-018-36687-4

**Published:** 2019-01-23

**Authors:** V. Demir, M. Akbulut, D. T. Nguyen, Y. Kaneda, M. Neifeld, N. Peyghambarian

**Affiliations:** 1 Present Address: ASML Inc, 77 Danbury Road, Wilton, Connecticut 06897 USA; 2 College of Optical Sciences, University of Arizona, Tucson, AZ 85721 USA; 3 Present Address: Corning Research and Development Corporation, Corning, NY 14831 USA

## Abstract

For the first time, we demonstrate injection locking and single frequency operation of a multi-core Yb-doped phosphate fiber laser (MCF). The 19 MCF laser cores operated in CW mode at 1030 nm. Each laser core was locked to the frequency and polarization of the single-frequency master laser, and produced milliwatts of power with similar lasing thresholds. The pump beam was homogenized with a simple technique to increase uniform lasing behavior of the cores. This behavior was verified using a MCF laser model developed in-house. This unique MCF laser can be useful for applications of coherent, coupled oscillator networks, for example in an all-optical coherent Ising machine configuration.

## Introduction

Optimization problems are ubiquitous across natural and social sciences, and the demand for their efficient solutions has been growing for significant impact almost in all areas of sciences and technologies, even in society^1–4^. The more challenging problems are classified as non-deterministic polynomial time (NP)-hard or NP-complete. These are known to be difficult to solve for exact solutions as the time and cost for solving such problems exponentially increases with the size of the problem^5^. Conventional solutions involve implementing mathematical algorithms such as stimulated annealing using digital computers resulting in approximate or “good solutions”. It has been contemplated that coupled laser networks may enable accelerated solutions to the NP-hard optimization problems due to the similarities of laser network energy functions to the Hamiltonian functions for the complex problems. In 2014, the first optical coherent Ising machine (CIM) based on a “network of 4 degenerate optical parametric oscillators (DOPO)” has been demonstrated by Yamamoto, *et al*.^6^. Mapping of Ising model to the alternative “injection locked laser array” architecture have also been theoretically proposed for semiconductor lasers by Yamamoto, *et al*.^7^.

In order to increase the number of oscillators from 4 to 19 and beyond, we have started to implement a new injection locking, slave-master multi-core fiber laser approach for the CIM application. Injection locking of semiconductor laser arrays have been extensively studied since the 80 s, albeit generally in the reference frame of coherent combination and/or linewidth narrowing^8,9^. The required injection locking of multi-core fiber lasers has not been done before and particularly for the CIM application that the requirements are different. For CIM application, we need all the lasers to act independently so that they can be injected into each other for the nonlinear network energy function evolution. This is in addition to the requirement that all the laser network nodes of the machine have to lase in a single longitudinal mode and should be identical.

In our work reported here, we propose mapping of the Ising model to the coherent, coupled laser network formed by an injection locked, Yb-doped MCF laser as a variation of the architecture in^7,10^. We have selected MCF lasers due to our expertise in MCF fabrication, and the fact that MCF lasers are known to be compact, robust and scalable to hundreds of cores with many developing applications^11–15^. Other benefits of MCF lasers include reduced component count, simplified synchronization, and potential for coherent operation of the cores through evanescent coupling (although we purposefully avoid this functionality in our work since we want independent but degenerate lasers). In this paper, we discuss the first step towards an optical CIM, namely a 19-core, single-frequency Yb-doped MCF laser that operates at 1030 nm. Single-frequency and single polarization operation of all cores are achieved via an intra-cavity volume Bragg grating (VBG) and injection locking. We believe this is the first demonstration of such an MCF laser.

## Experiment Setup and Mcf Laser Model

**Figure 1:**
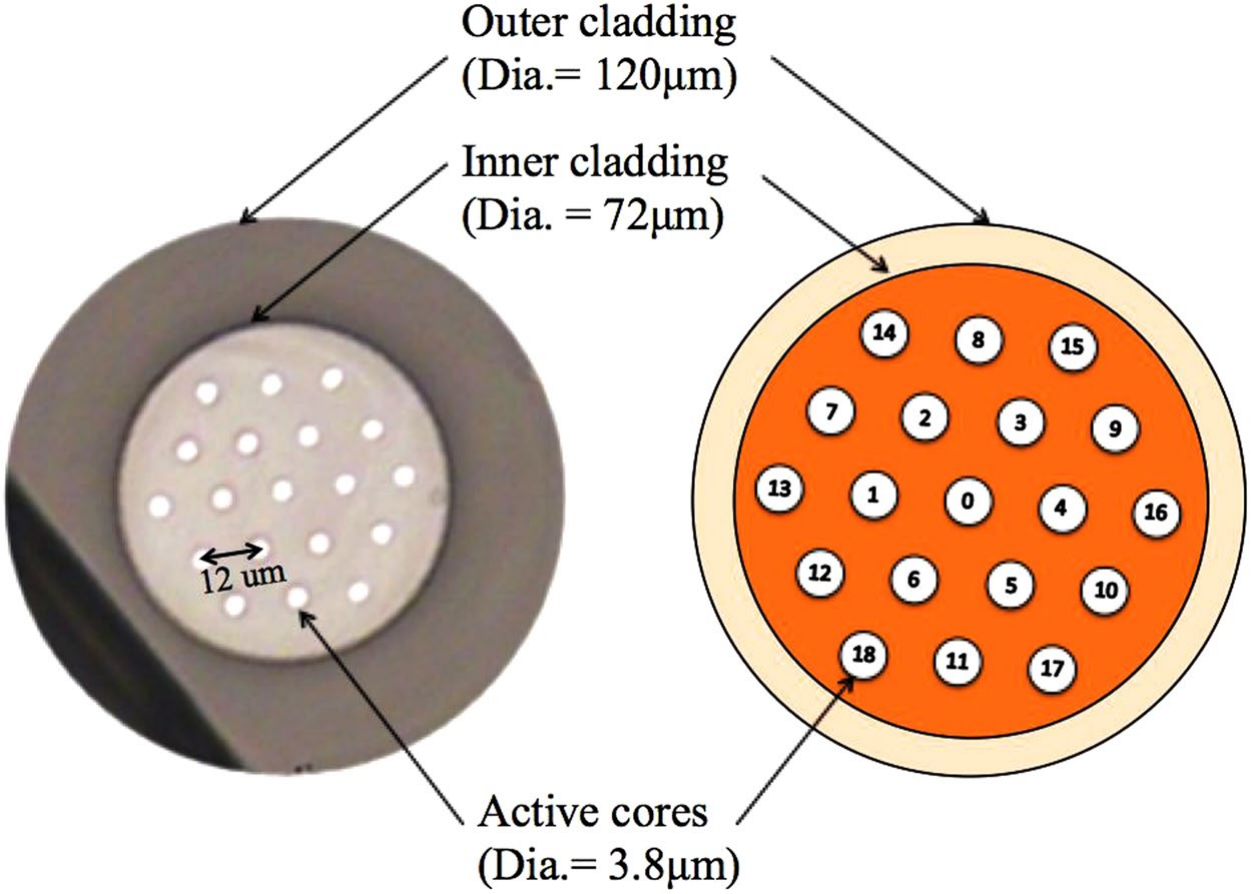
Cross section of 19-core Yb-doped double-cladding phosphate glass multicore fiber (MCF).

Figure 1 shows the cross section of the Yb-doped phosphate MCF. The fiber has an all glass double-cladding design for cladding pumping, with 19 equivalent cores placed in a honeycomb format. The fiber is made by NP Photonics Inc. from proprietary phosphate glasses and doped in the core with 6 wt% Yb_2_O_3_. The individual core diameters are 3.8 μm with core-to-core spacing of ~12 μm, and core numerical aperture (NA) of 0.15. The inner and outer cladding diameters are 72 μm and 120 μm, respectively. The NA of the inner cladding is 0.23, which is ideally suited for fiber coupled 975 nm pump lasers. The Yb-doped MCF is intentionally designed to have high gain and weak evanescent coupling among the cores for the signal wavelength of 1030 nm. Especially, in the short fiber lengths of 5–6 cm used in our experiments, the couplings are negligibly weak, and therefore we do not expect the supermodes which are the modes of coupled cores in strong coupling regimes. The MCF was mounted to a metal block with a V-groove by applying a thermally conductive compound. Less than one mm of the fiber tips were allowed to hang out of both sides of the metal V-groove in order to reduce the vibration of the fiber tip due to airflow and improve cooling and stability of the laser.

For pumping of the Yb-doped MCF, a fiber-coupled multi-mode pump laser diode at 975 nm was used. Up to ~4.1 W power from the pump laser fiber was free-space coupled to the inner cladding of the MCF fiber. The MCF fiber had one side flat-cleaved and serving as one of the cavity mirrors (~4% reflection), and the other side angle-cleaved to prevent instability due to feedback. For straightforward and stable injection locking, we have decided to build the free-running MCF laser cavities to operate close to the master laser frequency, and with as small number of modes as possible^14^. For this purpose, we have acquired a VBG with 1030 nm central wavelength, <0.1 nm FWHM and >90% efficiency (made by OptiGrate Inc). The VBG was used as the other high-reflection (HR) cavity mirror, and the flat cleaved fiber facet was used as the low-reflection (LR) cavity mirror. The overall cavity length was ~45 cm (including the ~6 cm gain fiber), yielding a nominal ~310 MHz between cavity modes. In the free running regime, the laser longitudinal modes are mainly defined by the VBG reflection spectrum, which has a FWHM of 0.05–0.1 nm FWHM. This corresponds to 14–28 GHz of bandwidth at 1030 nm. Considering the nominal 310 MHz mode spacing, many random and dynamic longitudinal lasing modes exist for each slave laser. However we did not observe significant differences in the FSR of each slave laser modes (investigated using a free-space scanning Fabry-Perot interferometer). This is somewhat expected since all the slave laser cores reside in the same mechanical fiber. Therefore thermal fluctuations in the length of the MCF fiber affect all slave cores in a similar fashion. Once the master laser (ML) is injected, all the random longitudinal lasing modes collapse into a stable single-frequency.

**Figure 2:**
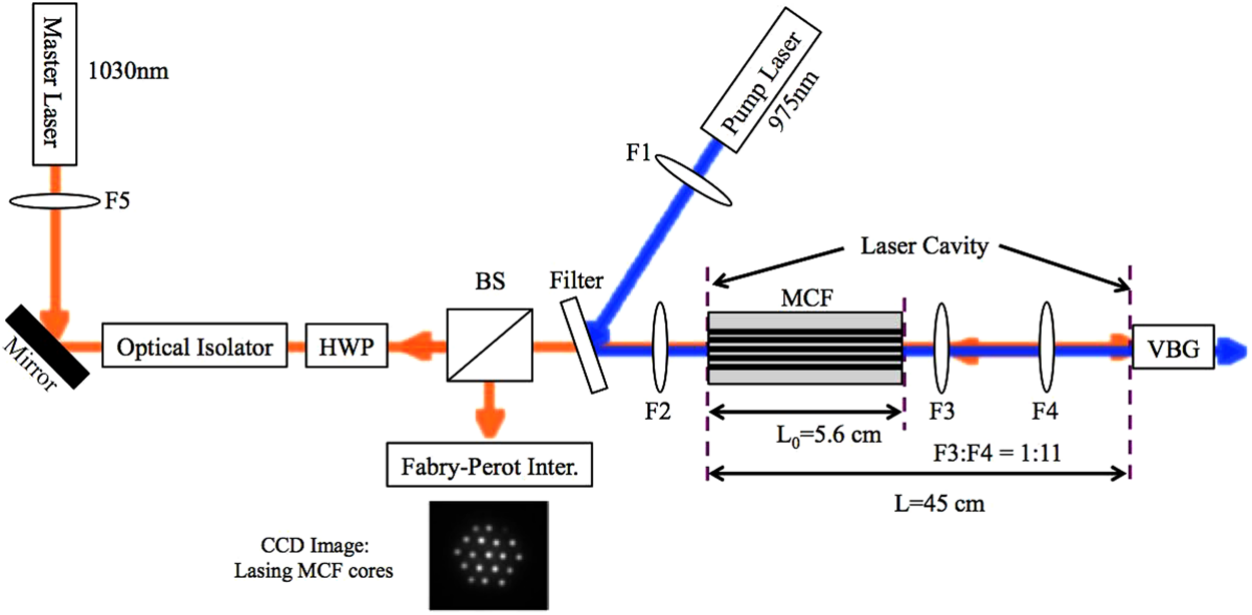
Experimental schematic of master/slave laser configuration. MCF: Multicore fiber laser; HWP: Half wave plate; Filter: Passes 1030 nm, reflects 975 nm; BS: Non-polarizing beam splitter; VBG: volume Bragg grating.

The ML source was a single frequency tunable diode laser at 1030 nm (Toptica DL-100) that was amplified up to ~200 mW of average power using a low-noise Yb-doped fiber amplifier. This increased power from the ML was required since the ML output was imaged to the inner cladding of the MCF, therefore injecting every core with a fraction of this power (less than 1 mW per core). The imaging lenses were carefully chosen and aligned for low coupling losses and aberrations. An optical isolator was placed after the ML to prevent any destabilizing feedback from the MCF laser to the ML. A half-wave plate enabled the selection of any linear polarization for injecting the MCF cores. This in turn enables us to injection-lock the MCF laser cores to any desired polarization state. The full experimental setup can be found in Fig. 2.

**Figure 3:**
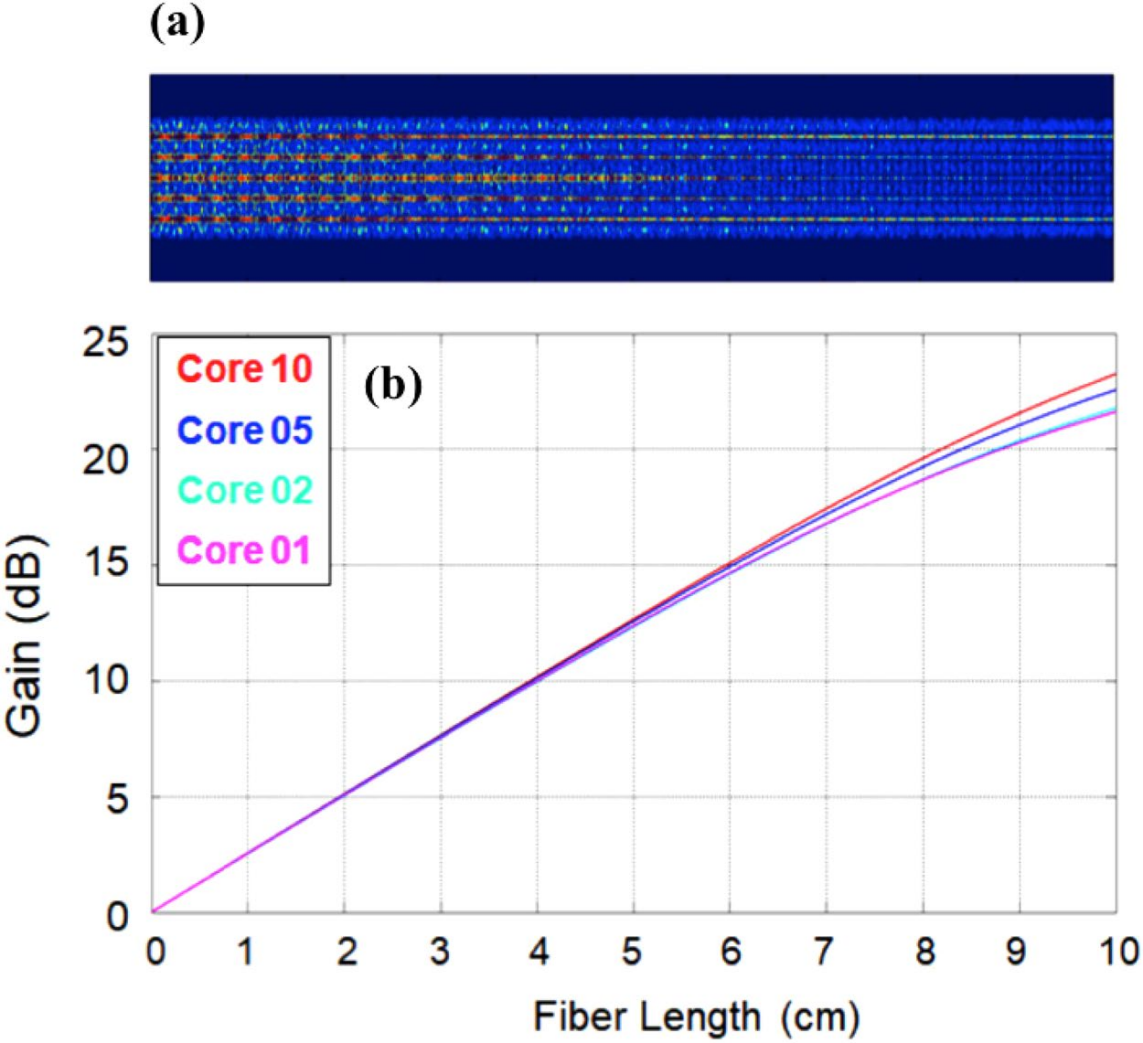
(**a**) Transverse pump beam profile vs. fiber length; (**b**) and gain of MCF laser with pump power of 4 W.

Uniform multi-laser behavior and injection-locking requirements require each MCF core to lase with similar threshold and efficiency. In order to understand and optimize performance of MCF laser, we have simulated the transverse beam profiles across the fiber length and gain per MCF core (shown in Fig. 3(a,b)) using our own modeling software based on an effective beam propagation method^16^. Note that, BPM can describe multimode propagation of the multimode pump beam in multi-core fibers very well. The method only requires fundamental parameters of the system such as core sizes, core and cladding refractive indices and positions of each waveguide. It doesn’t require any pre-calculated parameters such as propagation constants, numerical aperture (NA) or overlap factors, etc. Next, we present a brief description of the BPM for simulating cladding-pumped Yb-doped MCF in this work. The paraxial wave equation for the slowly varying electric field envelop of light propagating along the waveguide can be written as^16^:

1 $$\frac{d}{dz}\vec{E}(x,y,z)=(\widehat{D}+\widehat{V})\vec{E}(x,y,z)$$

where the operators $\widehat{D}$ and $\widehat{V}$ are given by $\widehat{D}=\frac{{\rm{i}}}{2k}(\frac{{\partial }^{2}}{\partial {x}^{2}}+\frac{{\partial }^{2}}{\partial {y}^{2}})$ and $\widehat{V}=[{\rm{i}}k{\rm{\Delta }}n(x,y,z)-\alpha (x,y,z)]$.

In the operators,

$$k={n}_{b}{k}_{0}=\frac{{n}_{b}\omega }{c}=\frac{2\pi {n}_{b}}{\lambda }$$

where *n*_*b*_ is the background or reference refractive index and *λ* is the free-space wavelength, ${\rm{\Delta }}n=n(x,y,z)-{n}_{b}$ is the refractive-index profile relative to reference refractive index, and $\alpha (x,y,z)$ is the power absorption/loss of the waveguide.

A small propagation step is implemented using the following approximation:

2 $$\vec{E}(x,y,z+{\rm{\Delta }}z)=\,{e}^{(\widehat{D}+\widehat{V}){\rm{\Delta }}z}\vec{E}(x,y,z)\approx {e}^{\widehat{D}{\rm{\Delta }}z/2}{e}^{\widehat{V}{\rm{\Delta }}z}{e}^{\widehat{D}{\rm{\Delta }}z/2}\vec{E}(x,y,z)$$

where ${e}^{\widehat{D}{\rm{\Delta }}z/2}$ means take a half step of diffraction alone, and ${e}^{\widehat{V}{\rm{\Delta }}z}$ means take the whole step of linear propagation alone. This calculation is third-order accurate in the step length and requires that the change produced by each step is small compared to unity. Equation (2) can be solved very effectively by a fast Fourier transformation (FFT). Details of the calculation method are presented elsewhere^16^. It is worth to note, that the most difficult problem of cladding-pumped fiber amplifiers is how to accurately describe multimode beam propagation of pump in fiber amplifiers. The main reason for the difficulties is the combination of multimode propagation of the pump beam in complicated fiber structures such as cladding shapes, multiple cores, and the amplification processes. In our simulation method, the rate equations are solved numerically giving accurate solutions $\alpha (x,y,z)$ for the pump absorption in all cores for BPM equation (1). The profile of pump beam that couples to each core along the fiber length can be obtained by BPM. Incorporating the pump profiles into propagation equation of signals for all cores, all characteristics of the amplifiers such as gains, noise figure in all cores of MCF^16^. The simulation results are in very good agreement with experiments having many different structures and also different concentrations of Er/Yb, as presented in our previous publications^16^.

Based on these simulations, we have optimized the length of the MCF for uniform lasing. It was found that 5–6 cm the 19-core MCF yielded uniform gain across the 19 cores, and gains are high enough for all cores to lase even without external mirrors (both ends of the fiber flat cleaved). As described earlier, the fiber cores are highly doped with Yb concentration (6wt %) so that single-pass gains for the low signal regime can reach more than 15 dB at 6 cm fiber length, high enough for lasing without external feedback. Note that, the reflection at the interface between phosphate glass and air is about ~13 dB. The final MCF length that was used in the experiment was ~5.6 cm. The operation of MCF in such a short fiber length has two folds: (i) the gains are highly uniformed for all cores, and (ii) crosstalk among the cores is negligible. It is worth to stress here that we intentionally design the fiber that the signal is strongly confined in the cores so that crosstalk due to evanescent couplings among the cores are negligible in short distance. Because of negligible couplings, all cores operate as independent laser cavities, and there is no super-mode effect. As can be seen in this paper, our experiments using 5.6 cm Yb-doped MCF achieved not only uniformed gains but also lasing without external feedback device.

**Figure 4:**
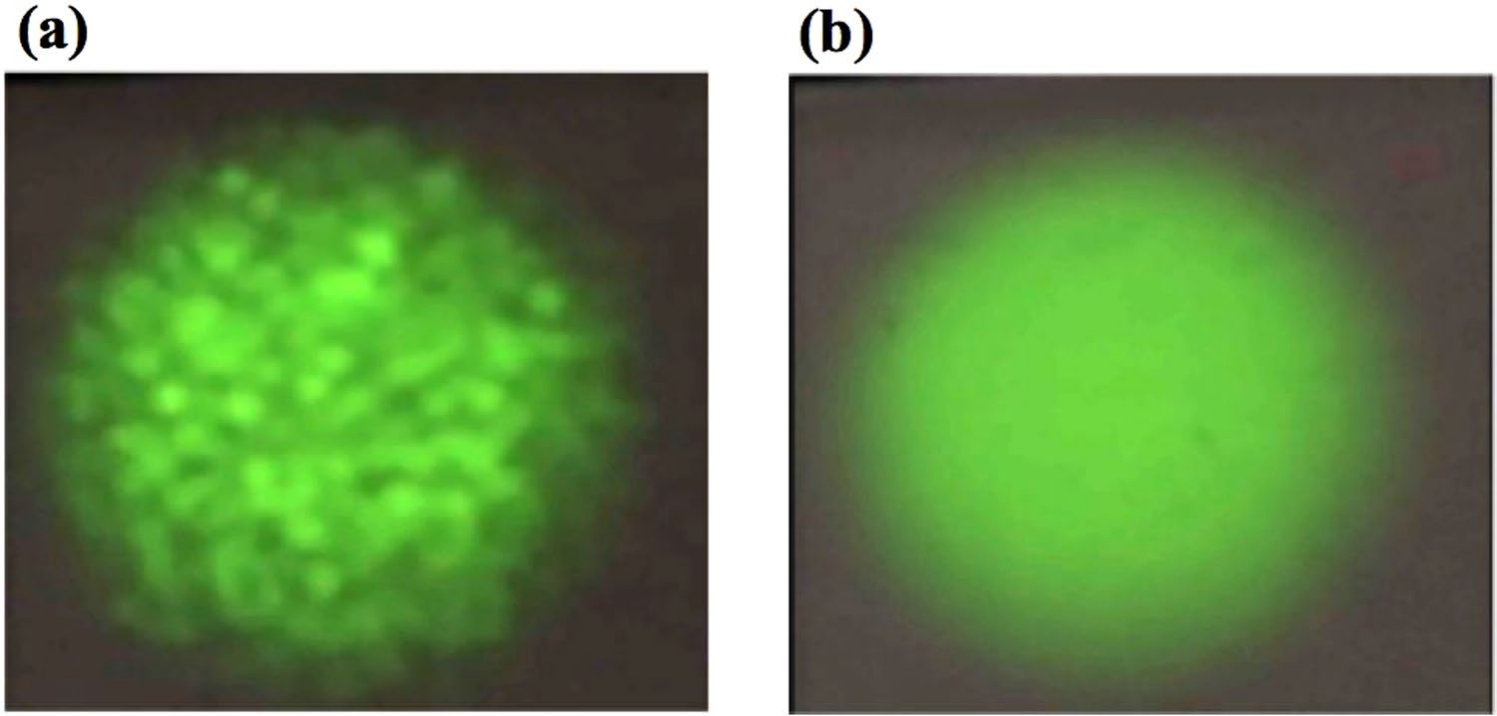
Pump beam profile snapshot (**a**) before and (**b**) after homogenization.

In addition to MCF fiber length, pump beam homogeneity was another critical parameter for lasing uniformity. The multi-mode pump diode laser had an output fiber with a core size of 105 µm and NA of 0.22. The pump beam from this fiber was observed to have randomly moving speckle pattern therefore causing non-uniform lasing thresholds in the cores. In order to eliminate this effect, first a commercial mode-scrambler that uses fiber-bending was utilized, however this caused too much pump power loss. Next, a custom vibration setup was designed and built in house, where the pump diode laser delivery fiber was gently shaken by a fast piezoelectric transducer. We picked a sinusoidal vibration pattern with a period of ~80 μs. Since this is much faster than the Yb lifetime of ~1 ms in the fiber, the MCF cores effectively see a uniform, flat top pump beam profile as shown on a ~1 ms response time CCD camera as shown in Fig. 4(a,b).

## Results

**Figure 5:**
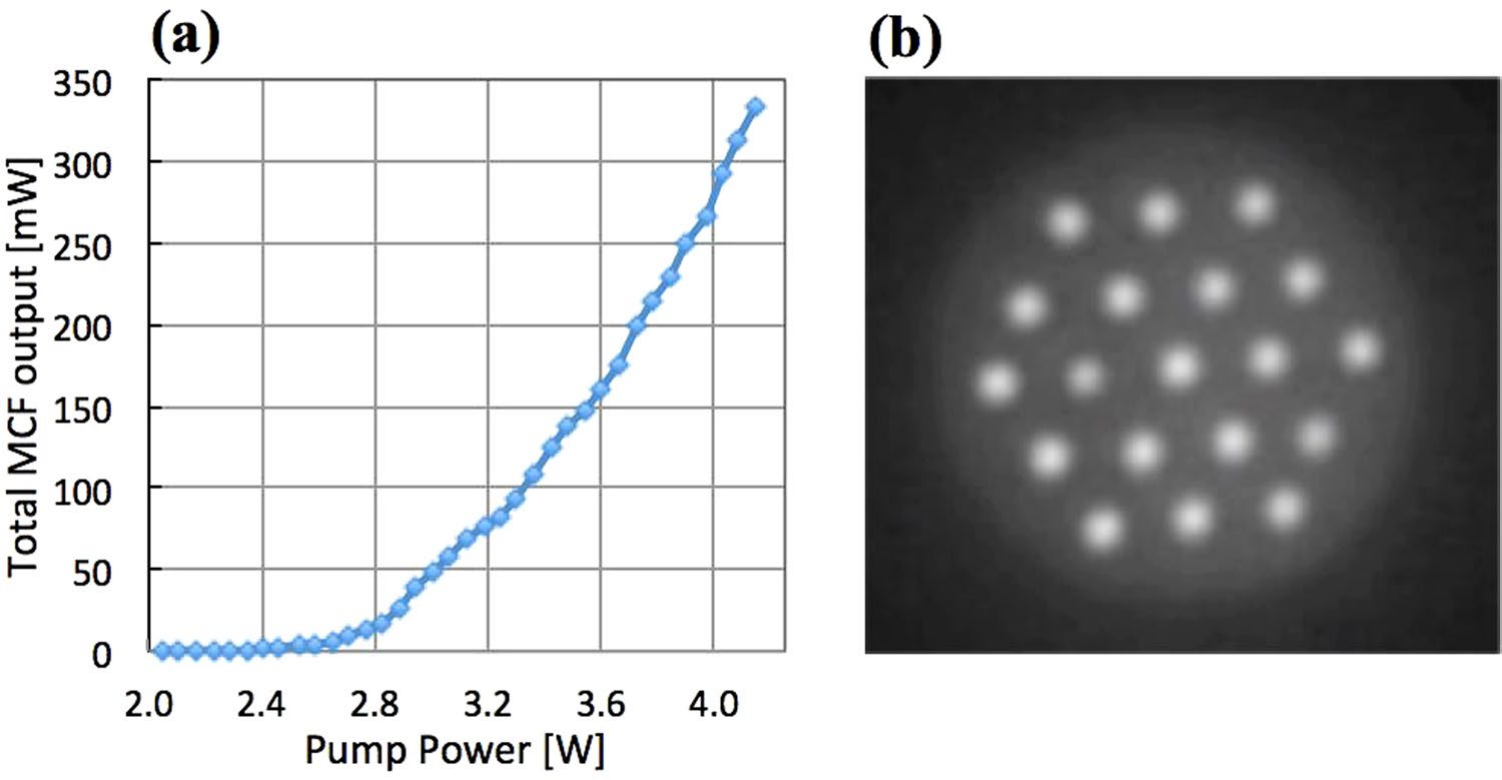
(**a**) Total free-running laser emission from the MCF; (**b**) The MCF cross-section imaged by a CCD camera.

The free-running MCF laser pump power vs. total signal power, and the relative intensities of the cores captured with a CCD camera image shown in Fig. 5(a,b), respectively. The pump power threshold for lasing was ~2.5 W and all of the cores started lasing at a pump power level of ~3.6 W. The top corner cores of the MCF are shown lasing with lower power in this picture due to non-optimal alignment of the pump beam.

**Figure 6:**
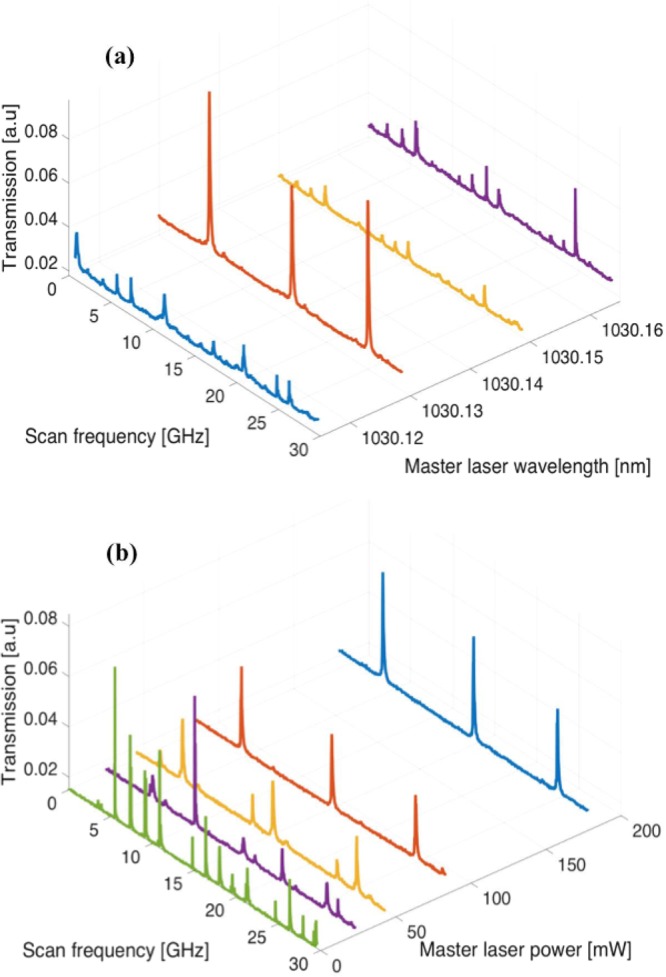
(**a**) Establishing the injection locking with ML tuning; (**b**) injection power optimization for the central core.

An initial investigation was conducted to find the optimal ML wavelength that would be close to one of the strong MCF cavity modes. For this purpose, we kept the MCF cavity stable while we tuned the ML frequency at ~500 MHz steps (~0.018 nm). Output of the MCF laser spectrum for the central core was measured through a free-space scanning Fabry-Perot interferometer. The results are shown in Fig. 6(a), where more than one free spectral range (FSR) can be seen. Due to the narrow bandwidth of the VBG, we were only able to find one strong mode that is close to the ML frequency. This exercise was also conducted to gauge the feasibility of cavity-locking using the ML frequency tuning feature (cavity-locking is briefly discussed towards the end).

Additionally, the ML power was tuned from 0 to 200 mW to find the suitable injection power range for the central core as shown in Fig. 6(b). Stable injection locking for the central core was observed for ≥100 mW input power. However, due to the Gaussian nature of the ML injection beam, the off-center cores requires more power for stable injection-locking. Therefore, the ML was operated at ~200 mW. It was also verified that, at the main output of the MCF laser (as shown in Fig. 2), all the cores followed the polarization state of the ML input. This input polarization could easily be varied via waveplates. The secondary output of the laser through the VBG exit port did not exhibit single polarization behavior.

**Figure 7:**
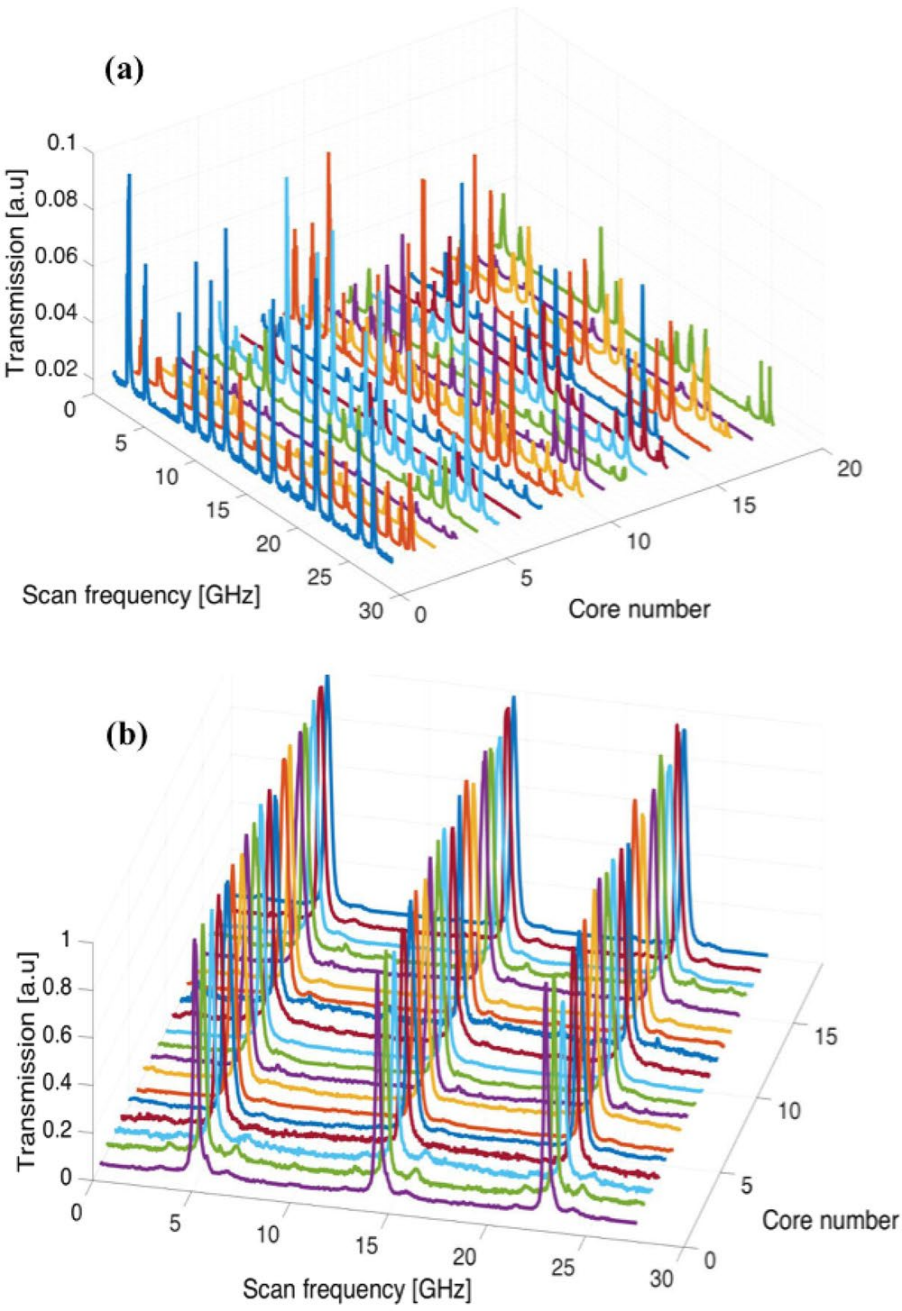
Transmission of (**a**) self-oscillating and (**b**) injection locked MCF laser cores to a master laser through scanning Fabry-Perot for 3 FSR.

The injection locking quality of each MCF laser core was investigated similarly to the central core with the help of the scanning Fabry-Perot interferometer. A CCD camera was also used to observe changes in the intensity of the locked slave lasers. Figure 7(a) shows the free-running MCF laser core spectra. As can be seen, the MCF lasers show multi longitudinal mode operation in the free-running mode. Upon injection of the ML into the inner cladding of the MCF with optimized power and wavelength as described above, the spectra of each MCF collapses to a single-frequency as measured through the scanning shown in Fig. 7(b).

**Figure 8:**
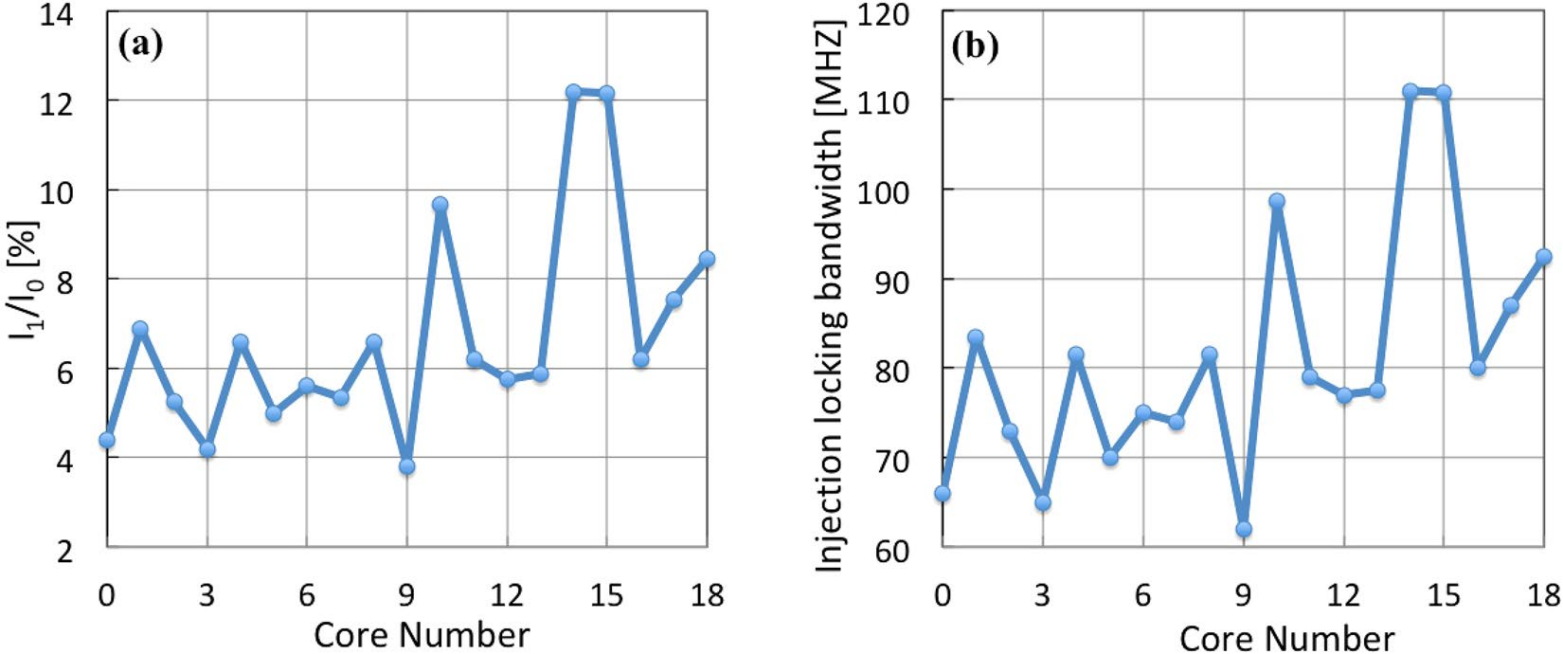
(**a**) Ratio of the powers of the master laser to self-oscillating MCF cores; (**b**) estimated full injection locking bandwidth for the MCF cores.

In order to estimate the injection locking bandwidth of each slave laser, we would need to calculate the injection power ratio for each core. For this purpose, a Gaussian model coupled with a geometrical filling factor was used. The calculated injection power ration (I_1_/I_0_) of ML to free-running MCF laser cores are shown in Fig. 8(a). Finally, the injection locking bandwidths were calculated and the results shown in Fig. 8(b). The variation in the injection locking bandwidth across the MCF cores is expected to be due to non-uniform master laser power and imperfect alignment of the master laser beam, pump beam, lenses and VBG in the cavity.

## Discussion and Future Work

The MCF cores stay injection-locked for a period of tens of seconds in an open laboratory environment Although this time frame may seem short, it is more than enough to conduct the Ising Machine experiment described in ref.^10^, which requires milliseconds for the system to reach the steady state solution (proportional to the lifetime of the Yb ion in the MCF). For longer-term stable injection-locking, one would be inclined to use a stronger ML injection, however this has previously been shown to degrade the phase information and increase instability based on detuning^17,18^. Therefore we did not choose this route. Rather, we have started investigating cavity locking of the ML and the MCF. This can be achieved two ways, either by modulating the MCF cavity length, or by modulating the ML laser frequency. We have first tried the MCF cavity modulation scheme. The ML output was sent through a free-space Phase Modulator (PM) for Pound-Drever-Hall (PDH) cavity locking technique before being injected into the MCF. We have mounted the VBG on a piezo translation stage to be able to feed the PDH error signal to the MCF cavity. In the second scheme, we fixed the MCF cavity, and tried to modulate the ML frequency by feeding the PDH error signal to the ML frequency modulation input. However, due to the slight cavity differences for each MCF core, we have not achieved stable operation at this time. We plan to continue the cavity locking experiments in the future with a modified PDH approach.

**Figure 9:**
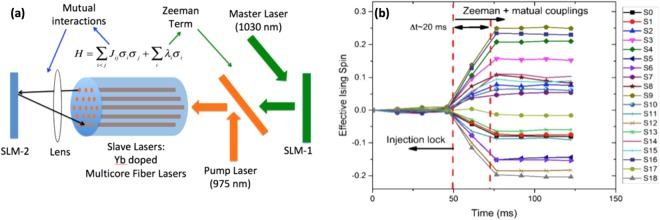
(**a**) Simplified schematics of Ising machine based on multicore fiber lasers, (**b**) Measured Ising states based on experimental Zeeman and coupling terms for the coherent Ising machine.

As we have mentioned in the introduction, the main application in mind for this unique MCF laser is to build an all optical Coherent Ising Machine based on ref.^7,10^. For this application, we have focused on obtaining independent yet degenerate slave laser cavities that can be coupled to each other. In this architecture, each MCF laser core that is lasing at the same frequency becomes a node of the Ising Machine. To convert our MCF laser in Fig. 2 into the optical Ising Machine, we have implemented two additions to the MCF laser setup as shown in Fig. 9a. The first is the insertion of a programmable Spatial Light Modulator (SLM) after the ML to be able to impart changes on the ML injection polarization in a core-to-core fashion. This enables us to program an initial condition (Zeeman term) for the Ising Machine. The second addition is another programmable SLM (using multiple beamsplitters) that is used to inject light from one MCF core to the others with variable amplitude and phase. This is the programmable cross-coupling terms for the Ising Machine. Once all the Ising terms are programmed, and the lasing started, the system will reach a steady state (in milliseconds) where the output polarization state of each slave laser is expected to indicate a solution of the Ising problem that was programmed.

Figure 9b shows the experimental results of the optical CIM. For this experiment, MCF was pumped at 3.6 W, and ~200 mW of power of ML was injected into the inner cladding of the MCF. The lasing threshold of the central core of the MCF was about 2.5 W. Initially, the Zeeman SLM was set at constant retardation, and cross-coupling section was mechanically blocked by a slow shutter (t < 50 ms in Fig. 7 left). Both the cross-coupling shutter and the Zeeman terms were abruptly turned on at t = 0 (t = 50 ms in Fig. 7 left), and the polarization states were recorded as a function of time until steady state was achieved (t > 75 ms in Fig. 9b). The tens of milliseconds convergence time for the polarization states were limited to the slow speed of the mechanical shutter and the camera readout time. Each slave laser core polarization was observed to move towards right-hand (positive spin) or left-hand polarization (negative spin) as expected. The signal-to-noise ratio was high enough to easily distinguish between positive and negative spins for each core.

## Conclusion

To conclude, we have demonstrated what we believe is the first injection-locked, single-frequency multi-core fiber laser at 1030 nm. Each of the 19 laser cores was locked to the frequency and polarization of the master laser, and produced milliwatts of power with mostly uniform lasing behavior. We have also developed a computer model that simulates and verifies the operation of the MCF laser. Finally, we have presented preliminary results in the application of this unique laser to an optical CIM. We plan to eventually use the MCF laser towards building a complete optical CIM setup.
